# Balance Performance in Autism: A Brief Overview

**DOI:** 10.3389/fpsyg.2018.00901

**Published:** 2018-06-05

**Authors:** John F. Stins, Claudia Emck

**Affiliations:** Department of Human Movement Sciences, Faculty of Behavioural and Movement Sciences, Amsterdam Movement Sciences, Vrije Universiteit Amsterdam, Amsterdam, Netherlands

**Keywords:** autism, postural control, anxiety, sensory integration, embodied cognition

## Abstract

Children with autism not only have limited social and communicative skills but also have motor abnormalities, such as poor timing and coordination of balance. Moreover, impaired gross motor skills hamper participation with peers. Balance control is interesting from a cognitive science perspective, since it involves a complex interplay between information processing, motor planning, and timing and sequencing of muscle movements. In this paper, we discuss the background of motor problems in children with autism, focusing on how posture is informed by sensory information processing. We also discuss the neurobiological basis of balance problems, and how this is related to anxiety in this group. We then discuss possible avenues for treatment of autism spectrum disorder (ASD) symptoms, especially as regards movement-related interventions. Finally, we present a theoretical outlook and discuss whether some of the symptoms in ASD can be understood from an embodied cognition perspective.

## Introduction

The control of everyday movements such as reaching, grasping, walking, gaze direction, etc., involves the concerted activity of neurocognitive processes, sensory processes, and reflexes. Ongoing movements have to be planned, initiated, guided, monitored, and adjusted to accommodate environmental contingencies. There is emerging insight that autism spectrum disorder (ASD) not only affects communication, cognition, mood and emotion, and behavioral regulation (American Psychiatric Association [APA], 2013), but also affects the control of movement. Even though sub-optimal motor skills are not considered a core feature of ASD, clinicians and researchers are well aware of motor deficits in ASD. For example, there is considerable overlap between the ASD phenotype and developmental coordination disorder (Sumner et al., 2016). The impact of motor difficulties in childhood can be severe, as it may contribute to reduced participation with peers during play and sports, and as a result may hamper social interaction and social development.

Some motor difficulties can be understood from a cognitive science perspective. The planning, coordination, and execution of motor actions involves the interplay between sensory processing, cognitive motor planning, and timing and sequencing of muscle activity patterns. There is evidence that information processing is affected in all these levels in ASD, and some current treatment approaches nowadays try to directly target these more basic cognitive processes. In this review, we focused on a very basic motor skill that is essential for the development of other gross motor skills, namely the act of upright standing, subserving postural control. Upright standing can be considered a tightly regulated, open- and closed-loop control process (e.g., Collins and De Luca, 1993). During upright standing, the body is in near postural equilibrium, but external and internal perturbations necessitate postural adjustments in order to prevent loss of stability. This process involves the integration of sensory inputs in order to accurately perceive postural orientation, and executing appropriate motor commands that restore postural equilibrium. Balance regulation is not purely reflex (spinal) driven, but higher centers are also involved, such as the motor cortex, basal ganglia, cerebellum, vestibular cortex, and brain stem. With respect to autism, several studies have found not only that postural control is compromised, but also that postural abnormalities are predictive of ASD symptomatology (examples below).

During school age, children with ASD display various difficulties with gross motor skills, such as running, jumping, and ball throwing (MacDonald et al., 2013). It is generally acknowledged that these skills are grounded in the (still developing) postural control system, especially in the age range 7–10 years (Mickle et al., 2011). Interestingly, worsened balance skills has been linked to anxiety, as evidenced by behavioral and neurological studies (Balaban and Thayer, 2001; Erez et al., 2004; Stins et al., 2009). A common feature of ASD is elevated anxiety (Kim et al., 2000; White et al., 2009; Ozsivadjian and Knott, 2011; Wijnhoven et al., 2018). Anxiety, in turn, could lead to activity avoidance. Also, anxiety could alter basic sensory processing, thereby affecting the manner in which sensory input is used to regulate balance (e.g., Horslen and Carpenter, 2011). Thus, a complex interaction between autism, anxiety, balance, and the development of gross motor skills is at stake. However, to our knowledge, the link between anxiety, ASD symptomatology, and postural control has not yet been systematically studied and clearly deserves attention.

Deeper insight into the severity, prevalence and neural origins of balance difficulties in ASD may help to understand and possibly interfere with a cascade of developmental problems. We first discuss the background of motor problems in children with ASD, with a specific focus on how posture is regulated. Next, we discuss movement-related interventions for treatment of ASD symptoms. Finally, we present a brief theoretical outlook and discuss whether ASD symptoms can be understood from an embodied cognition (EC) perspective.

## Balance Control in ASD

Balance abilities can be assessed either via standardized clinical tests to assess gross motor proficiency (including balance) in children, such as the Movement ABC (Henderson and Sugden, 1992) and the Test of Gross Motor Development (Ulrich and Sanford, 2000; Emck et al., 2011). Children with ASD score consistently lower on such standardized tests compared to controls (Berkeley et al., 2001; Green et al., 2009; Staples and Reid, 2010; Breslin and Rudisill, 2011; Whyatt and Craig, 2012; Liu and Breslin, 2013), and even to children with other kinds of (neuro)psychiatric disorders (Emck et al., 2009). For instance, we found that children with ASD showed the largest impairments in both locomotion and object control skills compared to the other psychiatric groups and typically developing children (Emck et al., 2011). In addition, the correlation between their scores for these subdomains was also significantly higher. It can be difficult to draw firm conclusions based on the available literature, due to considerable heterogeneity in sample sizes, ASD subtypes, age and IQ of the children, possible publication bias, and the dependent motoric measures. In that regard, the meta-analysis by Fournier et al. (2010) was a timely endeavor to gain insight into the motor coordination deficits in ASD. One of the key findings was that the motor deficiencies were especially prevalent as regards gross motor functioning, such as postural control. The authors also noted that a wide network of cortical and subcortical structures was implicated in the observed motor deficiencies. The authors concluded that “motor deficits are a potential core feature of ASD” (p. 1237), and that interventions should focus on improving (gross) motor skills. More recently, Moseley and Pulvermüller (2018) proposed a neurobiologically inspired model of suboptimal action-perception integration in ASD. According to these authors, ASD is characterized by a whole range of subtle motor control deficits (including postural instability), which may ultimately hamper normal cognitive and social development, for example, because there is less opportunity to explore and interact with the environment (see also Morris et al., 2015).

Balance proficiency can also be assessed by recording the center of foot pressure (COP) while standing on a force plate or a Wii balance board. Such devices record the time evolution of the point of application of the ground reaction force. The resulting time series yields insight into how an individual manages to regulate balance. During quiet upright standing, there is usually very little body sway in the left–right and fore–aft direction. However, when posture becomes more difficult, as when standing on one leg or standing with eyes closed, this leads to a significant increase in COP excursion, suggesting that greater effort is needed to prevent loss of postural stability.

Numerous studies have examined COP dynamics in ASD. We will not try to deal with all this literature, but here we note that the studies seem to fall in two broad categories. One set of studies examines to what extent the COP profile during quiet standing is predictive of several core ASD symptoms, thereby emphasizing the clinical utility of COP analysis. The second set of studies focuses on basic sensori-motor processing, and asks whether some sort of neural processing deficit may be responsible for aberrant COP profiles in ASD.

## Cop Patterns and ASD Symptoms

Travers et al. (2013) examined postural instability (more specifically, postural asymmetry) under various sensory and motor conditions during quiet standing. Their main finding was that adolescents with ASD displayed more instability than controls, but only when standing on one leg. The authors also examined the relationship between postural instability and repetitive behaviors, as assessed by a caregiver. Repetitive behaviors are characterized by repetition, rigidity, invariance, and inappropriateness, and interfere with adaptive functioning, and are considered one of the core symptoms of ASD (American Psychiatric Association [APA], 2013). It was found that postural asymmetry during two-legged standing was predictive of the presence and severity of repetitive behaviors^1^. A comparable finding was reported by Radonovich et al. (2013), who found that both the frequency and intensity of repetitive behaviors correlated with sway area (another commonly used metric of postural instability). However, the authors concluded that motor control impairments relate to a subset of ASD individuals, whose profile still needs to be determined. ASD is characterized not only by repetitive behaviors, but also by impaired social processing, also impacting postural control. Ghanouni et al. (2017) presented children with ASD and controls with social stimuli, namely pictures of neutral male and female faces (and an object), during quiet upright standing. The main finding was that children with ASD exhibited an increase in postural sway (root mean square and sway velocity) when viewing the faces. The authors presented several possible explanations for this finding, such as increased arousal and/or dual-tasking effects. A comparable paradigm was used by Gouleme et al. (2017), involving faces displaying various emotions. One of the findings was that children with ASD exhibited more sway (greater sway path length), especially when confronted with happy and sad faces. Additional recordings of eye movements revealed an aberrant pattern of fixations in various regions of the face. To sum up, two core symptoms of ASD, namely the presence of repetitive behaviors, and abnormal social processing, were evident in postural dynamics.

## Sensori-Motor Processing in Balance Control

In the study of Minshew et al. (2004), individuals with ASD (both children and adults) were measured while vision and/or ankle proprioception was manipulated. This was done by sway referencing the platform (i.e., changing the angle of the support surface). Compared to age- and IQ-matched controls, especially the sway referencing trials resulted in postural instability (peak-to-peak excursions of sway) in ASD. In our own study (Stins et al., 2015), we found that – at baseline – children with mild autism did not differ in their sway compared to controls. However, standing with eyes closed induced greater postural instability in the ASD group, especially in the medio-lateral direction. In the study of Doumas et al. (2016), both visual and proprioceptive information during quiet standing were temporarily made less accurate. Postural sway was recorded in a group of young adults with ASD and a group of controls. It was found that differences in postural instability (variability of sway and sway area) between the groups increased with the unreliability of sensory information. This suggests that individuals with ASD display “hyper-reactivity” to sensory disturbances. In the study of Chen and Tsai (2016), children with ASD were permitted to lightly touch a wall with their fingertip during quiet standing. The wall thus served as a spatial reference that could be used to regulate postural orientation, leading to reduced (more stable) sway. Interestingly, this reduction in sway was greater for children with ASD than controls. Thus, they benefited more from this tactile input to regulate balance.

These and many other studies show that sensory information processing, as well as the sensory control of balance, works in a sub-optimal manner in ASD. The meta-analysis of Lim et al. (2017) clearly delineated how sensory processing impairments negatively affect postural control in ASD. Overall, individuals with ASD exhibited more postural sway in response to almost all sorts of visual and somatosensory manipulations, and the authors suggested that the integration of information from different sensory channels into an appropriate motor response was hampered in this group.

## Movement Interventions

The observation of motor abnormalities has given rise to studies aimed at reducing symptoms via movement interventions. Underdeveloped motor skills may induce a pattern of movement avoidance, body weight gain, and reduced physical interaction with peers. As a result, the difference in motor competence with typically developing peers will increase, leading to the so-called “skill learning gap” (Wall, 2004). Psychomotor interventions can be used to counteract this negative self-sustaining cycle. Various studies have examined how motor activities such as running, swimming, horseback riding, cycling, strength and endurance training, etc., could reduce ASD symptoms. Indeed most studies reported an increase in social and motor competence, such as a reduction in aggression and stereotypical (repetitive) behaviors, better school performance, etc. The reader is referred to the following literature reviews (Lang et al., 2010; Sowa and Meulenbroek, 2012; Sam et al., 2015).

Since individuals with autism have reduced bodily awareness (see for instance Fiene and Brownlow, 2015; Asada et al., 2018), the therapeutic use of movement interventions should not only be geared toward increasing movement proficiency, but also to enhance body awareness, that is, reclaiming ownership of the own body in combination with becoming more responsive to external stimuli. The experience of body ownership is related to interoception, i.e., “the conscious perception of internal bodily cues such as heartbeat and breathing” (Schauder et al., 2015, p. 2). Likewise, Craig (2002) defined interoception as the sense of the physiological condition of the entire body, including the state of the internal organs. Importantly, the evaluation of changes in this state could serve as a basis for emotional experience, reminiscent of the James–Lange theory of emotion. The review of Critchley and Garfinkel (2017) clearly points to a causal role of interoception for emotional and motivational processes, and it could be the case that aberrant emotional processing in ASD in – in part – caused by abnormal interoceptive awareness in this group. Interoceptive ability is often assessed using a heartbeat counting task. With respect to ASD, the literature has yielded mixed results; Schauder et al. (2015) found no difference in accuracy of heartbeat counting between children with ASD and controls. In contrast, Garfinkel et al. (2016) found clear impaired performance on a similar task, leading them to propose therapeutic training to enhance interoceptive awareness in this group. Interestingly, Fiene and Brownlow (2015) reached the same conclusion, yet based on self-report measures (the Body Awareness Questionnaire).

Emck (2014) argued that a therapeutic intervention based on dance could be very promising, as not only this involves overall motor competence, but also it could increase relational awareness as regards facial expressions, adopting specific body postures in a social setting, etc. This is in line with the notion of Eigsti (2013) that it is important to counterbalance the weakened role of embodied processing in children with ASD by offering them tailored movement experiences. Similarly, Peper et al. (2016) considered ASD as lacking “bodily connectedness.” This term refers to movement patterns, especially of rhythmic nature, that help create and establish between-person social bonds. Especially, imitative behaviors and synchronized movements are of utmost importance in creating psychological connectedness. Indeed some preliminary studies are now underway that examine how a therapy based on dance (a very basic whole-body rhythmic activity) can help to establish a non-verbal communicative dynamic dyad with a therapist (e.g., Samaritter and Payne, 2017).

Given the role of anxiety and the balance problems in children with ASD, and the need for early intervention strategies to target anxiety in ASD (Wijnhoven et al., 2018), one might expect some literature about the efficacy of balance training. However, at present very little is known about the potential benefits of such interventions. As an example, Chelvadi et al. (2014) developed a 6-week balance training program for children with ASD, involving various balance challenges such a standing on foam. It was found that balance improved after the training. However, it is unknown to what extent these children also exhibited reduction in core symptoms, such as emotion regulation. Various interventions that are directly or indirectly affecting balance have been proposed, such as circus activities (Sahli et al., 2013) and martial arts (Fong et al., 2012). Bart et al. (2009) demonstrated that a program of balance training helped to reduce childhood anxiety and to increase balance confidence. These and other studies demonstrate the potential benefits and applicability of balance training, but at present, a large-scale well-designed (longitudinal) study with respect to ASD is lacking.

## Theoretical Outlook and Conclusion

We would like to briefly mention the theoretical perspective of EC, because of its relevance for understanding the contribution of balance problems with respect to the phenomenology of autism. Briefly, EC states that the mind is not merely connected to the body, but that cognition emerges as a complex interplay between body, brain and environment (e.g., Barsalou, 2008). Our physical abilities and body morphology co-determine how the mind works. In other words, the mind should not be thought of as a disembodied entity that works according to some abstract set of rules, but it is intimately connected with the sensori-motor system. Recent authors also consider ASD from an EC perspective. Notably, Eigsti (2013) claims that ASD emerges as a relative lack of embodiment, meaning that children with ASD are less able to access social representations due to impairments in sensory-motor integration. Children with ASD have reduced access to this embodied knowledge of social cues, as evidenced by reduced uses of bodily gestures and spontaneous facial mimicry (De Jaegher, 2013).

More recently, Moseley and Pulvermüller (2018) argued that circuits in the brain linking perceptual information and action form the cornerstones (carriers) of higher mental functioning, such as cognition and emotion. In ASD, this link is not fully developed, leading to “disembodied” pathways for information processing. This relative lack of embodiment manifests itself in phenomena such as clumsiness, language deficits, as well as social and cognitive deficits. Especially, early motor dysfunction – which involves problems in standing up and walking – could play a key role in the emergence of impairments in cognition and social processing in ASD. Consequently, we suggest that (subclinical) balance problems in ASD – as related to “particular ways of moving, perceiving and emoting” (De Jaegher, 2013, pp. 14) – may underlie the phenomenology and treatment of ASD.

The review of Moseley and Pulvermüller (2018) is worthwhile, as it tries to put EC on a solid neuroscientific basis (especially the mirror neuron system), thereby clarifying how an underdeveloped central nervous system may be responsible for a wide range of behavioral symptoms in ASD. We concur with Donnellan et al. (2013), who view behavior (both normal and aberrant) as emerging through a dynamical interaction between body, brain, and environment, against the backdrop of individual developmental and maturational trajectories. These theoretical insights may help to deepen our understanding of the complex and varied nature of ASD, and help to design novel therapeutic interventions, based on a solid scientific footing.

## Author Contributions

JS and CE conducted the literature review. JS drafted the manuscript. CE provided the critical revisions. Both the authors approved the final version of the manuscript for submission.

## Conflict of Interest Statement

The authors declare that the research was conducted in the absence of any commercial or financial relationships that could be construed as a potential conflict of interest.

## Footnotes

1 Interestingly, Rodgers et al. (2012) found a clear relationship between repetitive behaviors and anxiety, thereby underscoring the need to investigate the joint contribution of these markers to postural control in ASD.
